# Oral microbiota distinguishes patients with osteosarcoma from healthy controls

**DOI:** 10.3389/fcimb.2024.1383878

**Published:** 2024-07-11

**Authors:** Yu Chen, Chao Li, Xin Wang, Chun Lei Zhang, Zhi Gang Ren, Zhong Quan Wang

**Affiliations:** ^1^ Department of Pathogen Biology, Medical College, Zhengzhou University, Zhengzhou, China; ^2^ Department of Orthopaedic Surgery, The Affiliated Cancer Hospital of Zhengzhou University, Zhengzhou, China; ^3^ Department of Orthopaedic Surgery, Henan Provincial Chest Hospital, Zhengzhou University, Zhengzhou, China; ^4^ Department of Infectious Diseases, The First Affiliated Hospital of Zhengzhou University, Zhengzhou, China

**Keywords:** osteosarcoma, oral microbiota, characteristics, operational taxonomy units, diagnostic biomarker

## Abstract

**Objective:**

The human microbiota plays a key role in cancer diagnosis, pathogenesis, and treatment. However, osteosarcoma-associated oral microbiota alterations have not yet been unraveled. The aim of this study was to explore the characteristics of oral microbiota in osteosarcoma patients compared to healthy controls, and to identify potential microbiota as a diagnostic tool for osteosarcoma.

**Methods:**

The oral microbiota was analyzed in osteosarcoma patients (n = 45) and matched healthy controls (n = 90) using 16S rRNA MiSeq sequencing technology.

**Results:**

The microbial richness and diversity of the tongue coat were increased in osteosarcoma patients as estimated by the abundance-based coverage estimator indices, the Chao, and observed operational taxonomy units (OTUs). Principal component analysis delineated that the oral microbial community was significant differences between osteosarcoma patients and healthy controls. 14 genera including Rothia, Halomonas, Rhodococcus, and Granulicatella were remarkably reduced, whereas Alloprevotella, Prevotella, Selenomonas, and Campylobacter were enriched in osteosarcoma. Eventually, the optimal four OTUs were identified to construct a microbial classifier by the random forest model via a fivefold cross-validation, which achieved an area under the curve of 99.44% in the training group (30 osteosarcoma patients versus 60 healthy controls) and 87.33% in the test group (15 osteosarcoma patients versus 30 healthy controls), respectively. Notably, oral microbial markers validated strong diagnostic potential distinguishing osteosarcoma patients from healthy controls.

**Conclusion:**

This study comprehensively characterizes the oral microbiota in osteosarcoma and reveals the potential efficacy of oral microbiota-targeted biomarkers as a noninvasive biological diagnostic tool for osteosarcoma.

## Introduction

Osteosarcoma is the most common primary malignant tumor of bone, with a peak incidence in children and adolescents of approximately ~3–4.5 cases per million population worldwide (Mirabello et al., 2009; Beird et al., 2022). Although it can occur in any bone in the body, its mainly frequent sites are around the metaphysis and diaphysis of long bones such as the femur, tibia, and humerus (Cascini and Chiodoni, 2021). Additionally, it can arise in individuals concomitantly with a previous history of cancer as a secondary malignancy related to the bone (Ottaviani and Jaffe, 2009). Osteosarcoma is characterized by the presence of osteoid matrix or immature bone, which can lead to malignant progression and metastasis (Cascini and Chiodoni, 2021; Beird et al., 2022). In the past few decades, advancements in neoadjuvant chemotherapy and surgical techniques have significantly improved outcomes for patients with localized osteosarcoma, resulting in event-free survival rates exceeding 70%. However, the event-free survival rate drops to 20% in cases with metastasis at diagnosis or relapse (Gill and Gorlick, 2021). Notably, the key pathophysiological mechanism of osteosarcoma remains unclear. Furthermore, due to the absence of specific symptoms and the lack of reliable markers, most patients are often diagnosed in an advanced stage by the time they seek medical care for help, with poor prognosis. Therefore, it is important to explore novel diagnostic markers and efficacious novel therapies for osteosarcoma to improve the prognosis of osteosarcoma.

The human body harbors a diverse array of microorganisms that ensure vital functions for the host. These microorganisms can influence health, phenotype, and susceptibility to diseases by modulating physiological homeostasis, energy metabolism, and immune-related bioprocess (Eckburg et al., 2005; Rath and Dorrestein, 2012; Kundu et al., 2017; Levy et al., 2017; Dominguez-Bello et al., 2019). The oral microbiome is the second largest and most diverse microecosystem of up to approximately 1000 microbial species, which is crucial in maintaining oral as well as systemic health (Deo and Deshmukh, 2019). Emerging studies have shown that oral microbiota is associated with various diseases (Hayes et al., 2018; Radaic and Kapila, 2021; Hu et al., 2023; Lacunza et al., 2023), and can be used as a diagnostic tool for specific diseases or cancer, such as rheumatoid arthritis (Zhang et al., 2015) and pancreatic cancer (Vogtmann et al., 2020). Our previous research has verified the functional significance of the human tongue microbiota in COVID-19 (Ren et al., 2021) and SLE (Guo et al., 2023). However, the specific characteristics of the tongue-coating microbiota in patients with osteosarcoma have not yet been reported. In this study, we utilized 16S rRNA MiSeq sequencing technology to analyze the oral microbial signatures of osteosarcoma and focused on studying the association between osteosarcoma and oral microbiota. Our findings provide novel insights into the diagnostic and therapeutic potential for osteosarcoma.

## Materials and methods

### Study profile

This study was designed and performed in the light of the principle of PRoBE (prospective specimen collection and retrospective blinded evaluation) design, the Helsinki Declaration, and the Rules of Good Clinical Practice (Ren et al., 2019). It was approved by the Ethics Committee of the Affiliated Cancer Hospital of Zhengzhou University (2021-KY-0148–001) and the First Affiliated Hospital of Zhengzhou University (2021-KY-0716–003). All participants in this study approved and signed written informed consent before being recruited for the project.

A total of 147 samples were collected from the Affiliated Cancer Hospital of Zhengzhou University and the First Affiliated Hospital of Zhengzhou University between December 2020 and December 2022. This included 56 oral swabs of osteosarcoma patients (OS) and 91 oral swabs of healthy controls (HC) matched with osteosarcoma patients in terms of gender and age. The eligible patients fulfilled the following criteria: typical radiographic and histologic features of osteosarcoma of the extremity; newly diagnosed osteosarcoma; not having used antibiotics or probiotics in the 2 months before enrollment. Exclusion criteria included patients with other bone cancers or prior diagnosis with other tumors; non-extremity locations; non-compliance with National Comprehensive Cancer Network (NCCN) treatment guidelines, consumption of unhealthy substances including alcohol, cigarettes, and drugs; concomitant with previous history of infectious diseases, chronic diseases, oral mucosal diseases, gingival inflammation, dental diseases and throat diseases; and incomplete medical records. In addition, the relevant clinical data of participants including gender, age, body mass index (BMI), routine blood, liver function, kidney function, tumor site and size, Enneking stage, and metastasis were prospectively collected. Tongue-coating samples underwent 16S rRNA MiSeq sequencing. Following rigorous inclusion and exclusion criteria, 135 samples were included for further analysis.

### Oral sample collection

Before collecting tongue coating samples, each participant gargled twice with sterile water to maintain good oral hygiene. Then, a professional operator used a pharyngeal swab to sample the posterior middle to anterior middle area of the tongue coating. The collected specimen was immediately placed into a tube and transferred to a −80°C refrigerator.

#### DNA extraction, PCR amplification, and MiSeq sequencing

The DNA extraction process was performed by following the manufacturer’s instructions for bacterial DNA extraction using the E.Z.N.A. Stool DNA Kit (Omega Bio-tek, Inc., GA). The primers F1 and R2 (5’- CCTACGGGNGGCWGCAG -3’ and 5’-GACTACHVGGGTATCTAATC-C-3’) correspond to positions 341 to 805 in the *Escherichia coli* 16S rRNA gene were used to amplify the V3~V4 region of extracted DNA sample by PCR. The resulting amplicons from different samples were purified by Hieff NGS DNA Selection Beads (YeasenBiotech Co., Ltd., China). Subsequently, the products were indexed and mixed at equal ratios for sequencing by Shanghai Mobio Biomedical Technology Co., Ltd. using the Miseq platform (Illumina Inc., USA) according to the manufacturer’s instructions. Raw Illumina reads have been deposited in the European Bioinformatics Institute European Nucleotide Archive database under accession number PRJNA1016104.

#### Operational taxonomic unit clustering and taxonomic annotation

Raw sequencing data was processed by FLASH software (version 1.2.10) (Magoč and Salzberg, 2011). Operational taxonomic units (OTUs) were classified based on 97% identity using the UPARSE pipeline (version 11 http://drive5.com/uparse/) (Edgar, 2013) and OTUs were annotated at different taxonomic levels including phylum, class, order, and family and genus, using the RDP ClassifierV.2.626 (http://rdp.cme.msu.edu/) (Wang et al., 2007) against the SILVA^2^16S rRNA database.

### Bacterial diversity and taxonomic analysis

Bacterial richness and diversity were determined by a sampling-based analysis of operational taxonomic units (OTUs). The α diversity was evaluated by the Chao1, ACE, and Shannon indices which were calculated using mothur7 (version v.1.42.1). The β diversity was conducted using the R package (http://www.R-project.org/), and visualized through nonmetric multidimensional scaling (NMDS), principal component analysis (PCA) and principal coordinate analysis (PCoA). A heatmap of the identified key variables was plotted by the package pheatmap (http://CRAN.R-project.org/package=pheatmap).

To determine the relative differential abundance and the multivariable association between osteosarcoma patients and healthy controls, the PERMANOVA was used to identify features that differ in these groups. The specific characterization of the oral microbiota to distinguish taxonomic types was further analyzed by Linear discriminant analysis (LDA) effect size (LEfSe) method (http://huttenhower.sph.harvard.edu/lefse/) (Segata et al., 2011). The LEfSe method, based on the normalized relative abundance matrix, was used to pinpoint key bacteria in oral samples between osteosarcoma patients and healthy controls, according to the results of the Kruskal-Wallis rank sum test and Wilcoxon test (*p* < 0.05). The effect size of each feature was assessed by LDA (LDA score (log10) = 3 as the cut-off value) (Ling et al., 2014). Finally, Phylogenetic Investigation of Communities by Reconstruction of Unobserved States (PICRUSt) was utilized to assess the potential differences in metabolic pathways by comparing the 16S rRNA gene sequencing data against a reference database of microbial metagenomics including KEGG and MetaCyc database.

### Identification of the OTU biomarkers and construction of probability of disease

OTU biomarkers in the oral microbiome were selected using the Wilcoxon rank-sum test (*p* < 0.05) for further analysis. The optimal OTUs were identified through the random forest model with a fivefold cross-validation to construct a diagnostic model in the train set, effectively distinguishing between the OS group and HC group with high accuracy and stability (Heitner et al., 2010). The probability of disease (POD) index and Receiver operating characteristics (ROC) analysis were then performed to evaluate the quality of the classification models, with the area under the ROC curve (AUC) measuring the ROC effect (Robin et al., 2011). Moreover, the diagnostic efficacy of the diagnostic model was further validated in the test group.

### Statistical analysis

Statistical analyses were conducted using SPSS version 22.0 for Mac (SPSS, Chicago, Illinois, USA). Continuous variables between two groups were compared by Student’s t-test or Wilcoxon rank-sum test. Categorical variables between the two groups were compared by the χ^2^ test or Fisher’s exact test. Spearman rank correlation test was utilized for correlation analysis. Statistically significant differences were identified as *p* < 0.05 (two-sided).

## Results

### Study design and subject characteristics

**Figure 1:**
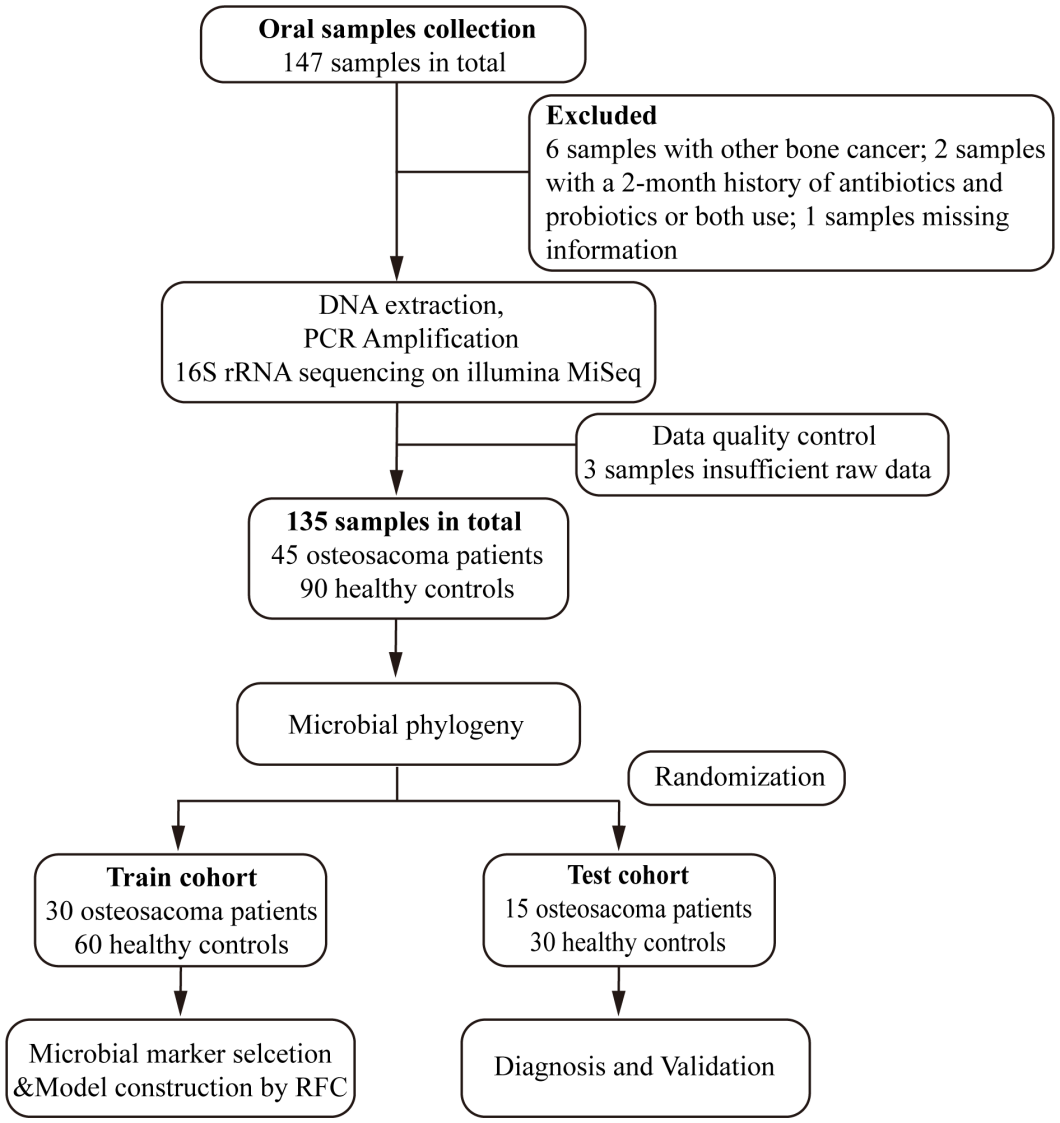
Study design and flow diagram. A total of 147 oral samples were collected. After a rigorous pathological diagnosis and exclusion process, 45 patients with osteosarcoma and 90 matched healthy controls were ultimately enrolled. Firstly, we analyzed the oral microbiota and identified key microbial markers in osteosarcoma patients and healthy controls. Then, all of the eligible participants were randomly divided into either the train set (30 OS verse 60 HC) or the test set (15 OS verse 30 HC). We constructed an osteosarcoma classifier by random forest model in the training group and validated the diagnostic efficacy in the test group. OS, osteosarcoma; HC, healthy control; RFC, random forest classifier.

Following strict pathological diagnosis and exclusion criteria, 45 patients diagnosed with osteosarcoma and 90 matched healthy controls were finally enrolled in the study. Initially, we characterized the oral microbiota and identified key microbial markers in both osteosarcoma patients and healthy controls. Then, all of the eligible participants were randomly allocated into a training set (30 OS verse 60 HC) and a test set (15 OS verse 30 HC). In the training group, prediction models were constructed based on the optimal OTUs and further validated for diagnostic efficacy in the test group (**Figure 1**).

The general characteristics of the overall participants, including the clinical data collected from medical records, are summarized in **Table 1**. Among the osteosarcoma patients, the mean age at diagnosis was 16.40 years (standard deviation 5.47) and 25 (55.56%) patients were male. Of the tumor sites, 41 (92.2%) were located in the lower extremities (femur, fibula, tibia), and 12 (7.80%) were found in the upper extremities (humerus, radius). The Enneking Stage included the severity, progression, and prognosis of osteosarcoma showing that 37 (82.22%) patients were categorized in stage II, and 8 (17.78%) patients were stage III. No significant differences existed in terms of age, gender, body mass index (BMI), and serum levels of white blood cells, red blood cells, hemoglobin, platelets, and albumin between the OS group and HC group, while clinically significant increases in the serum levels of alkaline phosphatase and serum creatinine were observed in the patient group, as detailed in **Table 1**.

**Table 1:** Demographics and clinical characteristics of participants in this cohort.

Clinical indices	Osteosarcoma (n=45)	Healthy control (n=90)	*p-*value
**Age (year)**	16.40 ± 5.47	17.14 ± 4.81	0.419
Sex			
Female	20(44.44%)	39(43.33%)	0.902
Male	25(55.56%)	51(56.67%)	
**BMI**	20.05 ± 4.32	20.43 ± 3.55	0.582
Tumor site			
lower extremity	39(86.70%)	−	−
upper extremity	6(13.30%)	−	−
**Tumor volume (cm^3^)**	303.71 ± 319.24	−	−
Enneking stage			
stages IIA-IIB	37(82.22%)	−	−
stages IIIA-IIIB	8(17.78%)	−	−
**WBC (10ˆ9/L)**	5.31 ± 1.40	5.39 ± 1.23	0.982
**RBC (10ˆ12/L)**	4.66 ± 0.48	4.67 ± 0.43	0.951
**Hemoglobin (g/L)**	140.93 ± 14.51	138.23 ± 15.91	0.162
**Platelet (10ˆ9/L)**	237.13 ± 41.54	258.01 ± 47.71	0.938
**Albumin (g/L)**	49.098 ± 2.63	48.89 ± 1.80	0.292
**Alkaline phosphatase (μ/L)**	262.27 ± 248.88	60.42 ± 15.33	< 0.001
**Serum creatinine (μmol/L)**	76.76 ± 10.51	69.91 ± 13.01	0.003

BMI, body mass index; WBC, white blood cells; RBC, red blood cells.

### The richness and diversity of oral microbiota increased in OS

**Figure 2:**
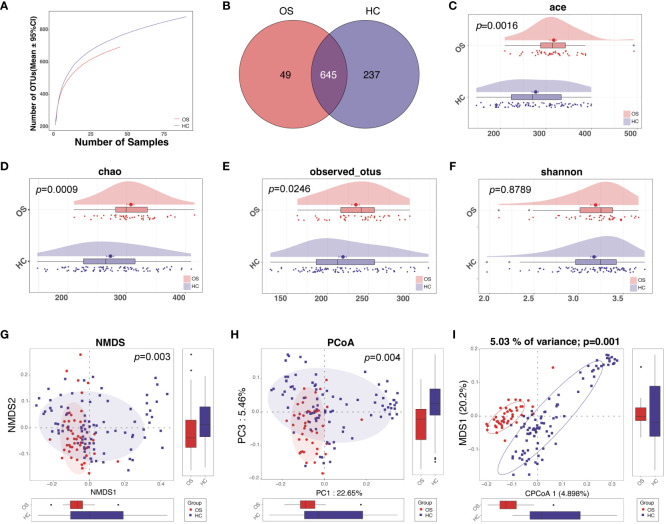
The diversity of oral microbiota increased in OS. **(A)** The Rarefaction analysis between the number of samples and OTUs. **(B)** A Venn diagram showed that 709 out of 832 OTUs were shared in both groups, while 49 OTUs were unique for OS and 237 OTUs were specific to HC. As measured by ACE **(C)**, Chao **(D)**, observed OTUs **(E)**, and the Shannon index **(F)**, the oral microbiota diversity was increased in OS (n = 45) versus HC (n = 90). The NMDS **(G)**, PCoA analysis **(H)**, and CAP analysis **(I)** showed that the OTUs distribution significantly differed in the two groups. OS, osteosarcoma; HC, healthy control; OUT, operational taxonomic unit; NMDS, nonmetric multidimensional scaling; PCoA, principal coordinate analysis; CAP, Canonical analysis of principal coordinates.

Rarefaction analysis was conducted to assess the species richness in each group. A flat curve indicates reasonable species abundance. The analysis of rarefaction demonstrated that the count of OTU richness basically approached saturation in each group, and it was significantly decreased in osteosarcoma (n = 45) compared to healthy controls (n = 90) (**Figure 2A**; **Additional File 1: Figure S1**). A total of 931 bacterial OTUs were identified across the entire cohort, with 645 OTUs shared by the two groups. Among these, 49 OTUs were unique for osteosarcoma and 237 OTUs were specific to healthy controls (**Figure 2B**).

We further tested whether the oral microbial diversity differed across multiple variables between OS and HC. For alpha diversity, there were significant differences between the two groups, with greater richness in the osteosarcoma group based on the abundance-based coverage estimator (ACE) indices, the Chao, and observed OTUs (**Figures 2C–E**; **Additional File 2: Data S1**). The Shannon index as one of the community diversities displayed a slight increase in the osteosarcoma tongue coating microbiome compared to healthy controls, although this difference was not statistically significant (**Figure 2F**). Beta diversity analysis revealed microbiome space between samples, which determines the distinct distribution of microbial composition. The distribution of OTUs was found to significantly differ in the two groups calculated using NMDS, PCoA analysis, and CAP analysis (**Figures 2G–I**).

### Phylogenetic profiles of the oral microbiota in OS

**Figure 3:**
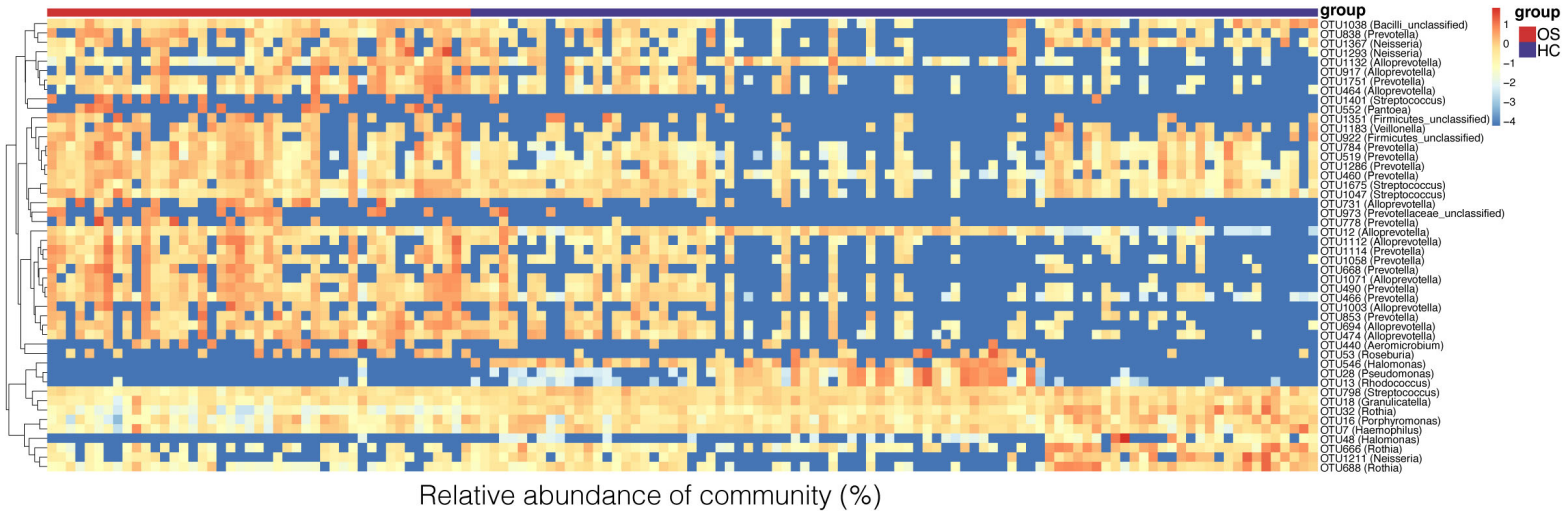
Heat map of the relative abundances of the discriminatory OTUs that drive the differences between OS (n = 45) and HC (n = 90). For each sample, the columns show relative abundance data of the differential OTUs listed on the right of the figure. The relative abundance of each OTU was used to plot the heat map (blue, low abundance; red, high abundance). Group information is shown above the plot: OS on the left with a red line. HC on the right with a blue line. Each row represents one OTU. OS, osteosarcoma; HC, healthy control; OUT, operational taxonomic unit.

To identify key OTU phylotypes in osteosarcoma, the taxa composition and variation of oral microbiota were analyzed in OS and HC based on the OTU annotations of each sample. A total of 48 distinguishing OTUs in the tongue-coating microbiota were identified as the key lineages in osteosarcoma patients and healthy controls (**Figure 3**, **Additional File 2: Data S2**). Of the discriminatory OTUs, 14 were decreased while 34 were increased in the oral microbiota of osteosarcoma patients compared to healthy controls.

**Figure 4:**
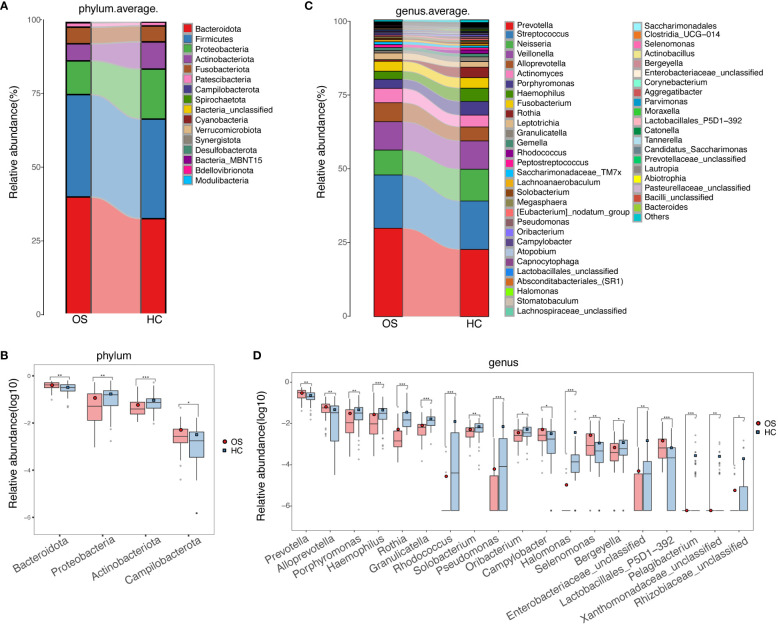
Phylogenetic profiles of the oral microbiota in OS. **(A)** The average compositions and relative abundances of bacterial taxa in both groups at the phylum level, respectively; **(B)** Significant differences among the abundances of discriminatory phyla between OS (red) and HC (blue); **(C)** The average compositions and relative abundances of bacterial taxa in both groups at the genus level, respectively; **(D)** Significant differences among the abundances of discriminatory genus between OS (red) and HC (blue). Significant differences by *p < 0.05; **p < 0.01 and ***p < 0.001. OS, osteosarcoma; HC, healthy control.

Relative abundances of taxa at phylum and genus levels were evaluated, and the average compositions and relative abundances of bacterial taxa in both groups were shown in **Figures 4A, C** (**Additional File 2: Data S3**, **S4**), respectively. Bacterial phyla of *Bacteroidetes*, *Firmicutes*, *Proteobacteria*, and *Actinobacteria* accounting for approximately 90%, were the main dominant populations in the two groups (**Figure 4A**; **Additional File 2: Data S5**). Compared with healthy controls, *Actinobacteriota* and *Proteobacteria* were decreased significantly in OS, while *Bacteroidetes* and *Campilobacterota* were increased (**Figure 4B**; **Additional File 2: Data S5**). There was no significant difference in terms of *Firmicutes* between the groups. Correspondingly, at the genus level, 14 genera including *Rothia*, *Halomonas*, *Rhodococcus*, and *Granulicatella* were reduced remarkably, whereas 5 genera including *Alloprevotella*, *Prevotella*, *Selenomonas*, and *Campylobacterota* were enriched in OS versus HC (**Figure 4D**; **Additional File 2: Data S6**). Additionally, bacterial differences at the class, order, and family levels in the two groups were also compared and shown in the **Additional File 1: Figures S2**-**S4** and **Additional File 2: Data S7**-**S9**.

### Key bacteria and microbial functions related to OS

**Figure 5:**
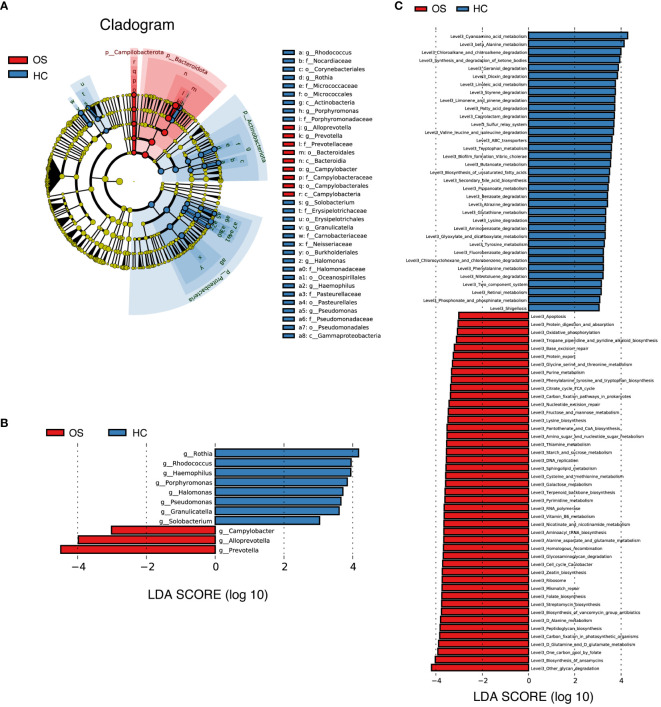
Key bacteria and microbial functions related to OS. **(A)** Cladogram generated by the LEfSe method indicating the phylogenetic distribution of oral microbiota associated with OS (red) and HC (blue); **(B)** LDA scores showed the significant bacterial difference between the OS and HC; **(C)** Prediction of the key functional and metabolic pathways between in the two groups. OS, osteosarcoma; HC, healthy control.

We further compared the oral microbiota using LEfSe to identify specific bacterial taxa and predominant bacteria associated with the alteration of oral microbiota between OS and HC, which implies the differences in oral microbiota in osteosarcoma patients. Based on the linear discriminant analysis (LDA) selection, a cladogram representative of key oral microbial structures and their major bacteria showed the greatest differences in taxa between OS and HC (**Figure 5A**; **Additional File 2: Data S10**). At the genus level, the tongue-coating microbiota within osteosarcoma patients was characterized by the preponderance of 3 genera including *Prevotella*, *Alloprevotella*, and *Campylobacter* (LDA score (log10) > 3). However, 8 genera including *Rothia*, *Rhodococcus*, *Halomonas*, and *Porphyromonas* were significantly enriched in the healthy controls group (LDA score (log10) > 3). The result is shown in **Figure 5B** (**Additional File 2: Data S11**).

Additionally, the relative alterations of major functional and metabolic modules concerning the different groups were demonstrated in **Additional File 1: Figure S5**, indicating the differences in functional patterns within osteosarcoma patients compared to the healthy controls. KEGG pathway/module analysis at level 3 (**Figure 5C**; **Additional File 2: Data S12**) demonstrated that 45 predicted microbial functions, including other glycan degradation, biosynthesis of ansamycins, one carbon pool by folate and D-Glutamine and D-glutamate metabolism were enriched in OS group, whereas 35 functions including cyanoamino acid metabolism, beta-alanine metabolism, chloroalkane and chloroalkene degradation and synthesis and degradation of ketone bodies were significantly abundant in HC (all *p* < 0.05, LDA score (log10) > 3).

Furthermore, Spearman’s correlation analysis was utilized to explore the relationship between the oral microbiome and clinical data of osteosarcoma. Nine OTUs were found to be related to six clinical indicators, including age, red blood cell count, hemoglobin, albumin, serum creatinine, and alkaline phosphatase (**Additional File 1: Figure S6**, **Additional File 2: Data S13**). Of note, alkaline phosphatase was negatively correlated with six genera including *Halomonas*, *Rothia*, and *Rhodococcus*, and positively correlated with one genus (*Alloprevotella)*. This suggested a potential interaction between oral microbiota and alkaline phosphatase that may be involved in affecting disease progression.

### Diagnostic model of the oral microbial OTUs-based markers for OS

**Figure 6:**
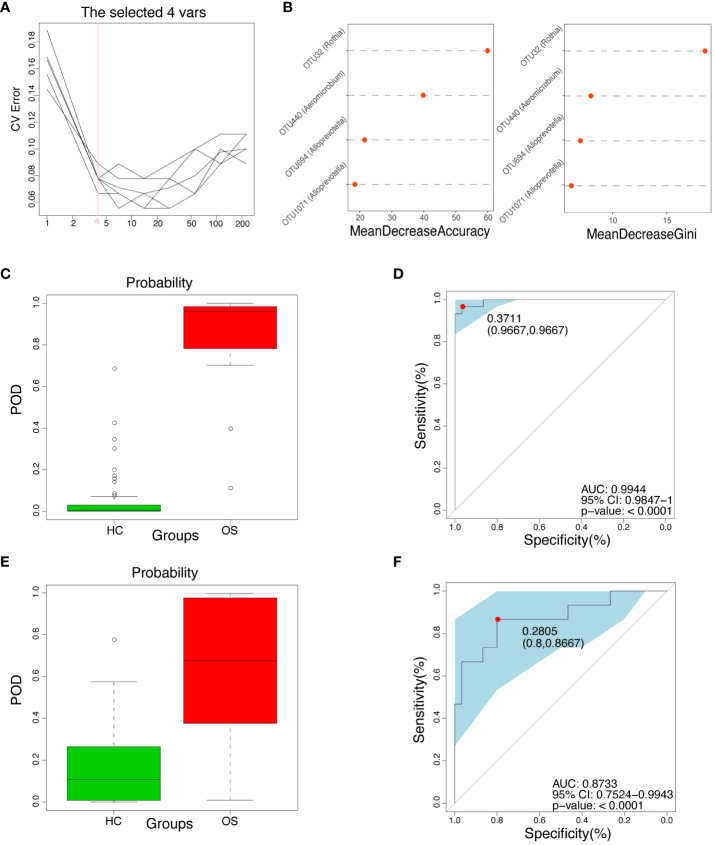
Diagnostic potential of oral microbial markers for OS. **(A)** Four OUTs were identified as the optimal markers set by the random forest model. **(B)** Importance distribution map of the selected microbial markers in the model. **(C)** The POD value was significantly increased in OS (n = 30) versus HC (n = 60) in the training cohort. **(D)** The POD value achieved an AUC of 99.44% (95% CI 0.9847–1.0000, p < 0.0001) in the training cohort. **(E)** The POD value was remarkably increased in OS (n = 15) compared with HC (n = 30) in the independent test cohort; **(F)** The POD value achieved an AUC of 87.33% (95% CI 0.7524–0.9943, p < 0.0001) in the independent validation cohort. OUT, operational taxonomic unit; CI, confidence interval; OS, osteosarcoma; HC, healthy control; POD probability of disease, AUC, area under the curve.

To explore whether oral microbial OTUs**-**based markers accurately distinguish OS from HC, a random forest prediction model was constructed using fivefold cross-validation in the training set (30 OS versus 60 HC). The results showed that four OTU markers, including OTU32 (*Rothia*), OTU440 (*Aeromicobium*), OTU694 (*Alloprevotella)*, and OTU1071 (*Alloprevotella)*, were identified as the optimal marker set (**Figures 6A, B**). The corresponding abundance of these four OTU markers in each sample was presented in **Additional File 2: Data S14**. Subsequently, the POD index was calculated using these four identified optimal OTUs in the training group. The POD value of osteosarcoma patients increased significantly compared to healthy controls, with an area under the receiver operating characteristic (ROC) curve (AUC) of 99.44% and 95% confidence interval (CI) of 98.47% - 100.00% (*p* < 0.0001) between the OS and the HC cohorts as shown in **Figures 6C, D** (**Additional File 2: Data S15**).

In addition, an independent validation group was utilized to confirm the diagnostic efficiency of the oral microbial marker model for osteosarcoma in the test set. The relative abundance of the four OTU markers in each sample within the test set was shown in **Additional File 2: Data S16**. **Figures 6E, F** (**Additional File 2: Data S17**) showed that the POD index of osteosarcoma patients was significantly increased compared with healthy controls, with the AUC value of 87.33% and 95% CI of 75.24% - 99.43% (*p* < 0.0001) in the test set. Importantly, the results suggested the potential of the oral microbial markers-based classifier model for diagnosing osteosarcoma in healthy individuals.

## Discussion

Osteosarcoma is the most common malignant bone tumor with an advanced tendency of invasion and metastasis (Beird et al., 2022). Although the therapeutic regimens (including surgical techniques, chemotherapy, and radiotherapy) have advanced significantly in the last few decades, the prognosis is still poor in osteosarcoma patients with metastases at diagnosis or relapse, resulting in a high mortality rate (Allison et al., 2012; Gill and Gorlick, 2021). This may be due to the rarity and heterogeneity of the tumor, as well as the lack of identified pathognomonic mutations and limited diagnostic markers (Davis et al., 1994; Kansara et al., 2014; Corre et al., 2020). Consequently, it is critical to identify reliable markers that can detect early osteosarcoma individuals to improve the prognosis of osteosarcoma.

The human microbiome has been defined as the new emerging “Hallmarks of Cancer” (Hanahan and Weinberg, 2011). Extensive studies have now uncovered the role of oral microbiota in the initiation, progression, and prognosis of multiple cancer types (Sepich-Poore et al., 2021; Wang et al., 2023), such as oral cancer (Zhang et al., 2019a), lung cancer (Hosgood et al., 2021), and colorectal cancer (Rezasoltani et al., 2022). However, the oral microbiota in osteosarcoma still needs to be further studied. In this study, we focused on analyzing the bacterial composition and functional changes in the tongue-coating microbiota of osteosarcoma patients compared to healthy controls. We identified the key microbiota and constructed a microbial classifier that achieved good diagnostic efficacy in distinguishing osteosarcoma from healthy controls in China. These findings suggested that the distinct oral microbiota profiles could potentially serve as a noninvasive biomarker for osteosarcoma.

The microbiota colonizing the tongue in healthy individuals was identified at the phylum level, including *Firmicutes*, *Bacteroidetes*, *Proteobacteria*, *Actinobacteria*, *Spirochaetes*, *Fusobacteria*, and *Synergistetes* (Sun et al., 2017; Li et al., 2021). Consistent with the results aforementioned, our study observed that *Bacteroidetes*, *Firmicutes*, *Proteobacteria*, and *Actinobacteria* were the predominant bacterial phyla in both osteosarcoma patients and healthy individuals. In comparison to healthy controls, *Actinobacteriota* and *Proteobacteria* were decreased significantly, while *Bacteroidetes* were increased in osteosarcoma. Furthermore, the different signature of oral microbiota in osteosarcoma patients was characterized by increased diversity and altered bacterial communities compared to healthy controls. Notably, the relative abundance of phylum *Bacteroidota* (*Alloprevotella*, *Prevotella*, and *Campylobacter*) exhibited the highest in osteosarcoma patients, followed by the unclassified phylum *Selenomonas*. Some of these bacteria are recognized as opportunistic pathogens that may pose a risk to the host. Specifically, *Alloprevotella* has been implicated in the development of oral cavity squamous cell cancer (Ganly et al., 2019) and the prognosis of colorectal cancer (Ge et al., 2020; Wang et al., 2021). *Prevotella* and *Selenomonas* genera are associated with severe early childhood caries (He et al., 2015; Zhang et al., 2019b), while *Campylobacter* is linked to malignant oral leukoplakia (Amer et al., 2017).

We further explored the relationship between oral microbiota and clinical indices in osteosarcoma. For example, *Alloprevotella* was positively correlated with alkaline phosphatase (r = 0.222, *p* = 0.009), indicating that the potential link between *Alloprevotella* and bone metabolism may be involved in the progression of osteosarcoma. Several clinical trials have reported significantly poorer overall survival in osteosarcoma patients with high levels of alkaline phosphatase than in those with low levels (Ferrari et al., 2001; Bacci et al., 2006). It is important to note that while our correlation analysis hints at a potential relationship between clinical parameters and oral microbiota. Whether there is a causal relationship and its specific mechanism, needs to be verified experimentally.

Recently, convincing studies have demonstrated that the oral microbiome is not only closely related to a variety of diseases but can also serve as a non-invasive diagnostic tool for specific diseases. Zhang et al. constructed an oral microbial diagnostic model and verified its diagnostic efficacy in rheumatoid arthritis (Zhang et al., 2015). Flemer et al. analyzed the alterations of the oral microbiome in colorectal cancer and developed a diagnostic model based on 16 optimal oral microbial markers, which achieved good diagnostic efficacy in distinguishing colorectal cancer from healthy individuals (Flemer et al., 2018). Our previous studies presented that oral microbial markers could be a potential non-invasive diagnostic tool for COVID-19 (Ren et al., 2021) and SLE (Guo et al., 2023). In this research, we established and validated a diagnostic model for osteosarcoma based on four optimal OTUs from the oral microbiota. This model effectively differentiated between osteosarcoma patients and healthy controls with an area under the curve of 99.44% in the training group and 87.33% in the test group, respectively. The oral microbial markers identified demonstrated promising diagnostic capabilities in discerning osteosarcoma cases. Overall, these findings underscored the potential utility of oral microbiota as a non-invasive diagnostic tool for osteosarcoma.

Despite the advantages outlined above, this study is limited by the scarcity of newly diagnosed osteosarcoma patients and challenges in obtaining an adequate sample size. Additionally, being a single-center study in China, the performance of the diagnostic model lacks validation across multiple regions. Furthermore, the mechanisms through which the oral microbiome contributes to osteosarcoma development have not been elucidated. These issues need to be investigated in further study.

## Conclusion

In summary, the study characterized the alteration of oral microbiota in osteosarcoma patients compared with healthy controls and identified key bacteria that distinguish osteosarcoma patients from healthy subjects. The results confirmed the association between oral microbiota and osteosarcoma. Notably, a new diagnostic model based on oral microbial signatures was established, thus providing a potential non-invasive diagnostic method for osteosarcoma. Therefore, the results suggested that oral microbiota could serve as targeted indicators for developing new diagnostic tools or treatment approaches for osteosarcoma in the future.

## Data availability statement

Raw Illumina reads have been deposited in the NCBI Sequence Read Archive (SRA) under the BioProject number PRJNA1016104.

## Ethics statement

It was approved by the Ethics Committee of the Affiliated Cancer Hospital of Zhengzhou University (2021-KY-0148-001) and the First Affiliated Hospital of Zhengzhou University (2021-KY-0716-003). The studies were conducted in accordance with the local legislation and institutional requirements. Written informed consent for participation in this study was provided by the participants’ legal guardians/next of kin.

## Author contributions

YC: Writing – original draft, Conceptualization, Formal Analysis, Investigation. CL: Resources, Writing – original draft. XW: Resources, Writing – original draft. CZ: Resources, Writing – original draft. ZR: Supervision, Writing – review & editing. ZW: Supervision, Writing – review & editing.

## Supplementary material

Supplementary Data Sheet S1 The detailed values of oral microbial diversity index and observed OTUs in the two groups (90 HC and 45 OS).

Supplementary Data Sheet S2 The relative abundance and distribution of the key 48 OUTs in the two groups (90 HC and 45 OS).

Supplementary Data Sheet S3 The composition and abundance of bacterial community at the phylum level in each sample (90 HC and 45 OS).

Supplementary Data Sheet S4 The composition and abundance of bacterial community at the genus level in each sample (90 HC and 45 OS).

Supplementary Data Sheet S5 The different degrees at the phylum level (p-value) between the two groups (90 HC and 45 OS).

Supplementary Data Sheet S6 The different degrees at the genus level (p-value) between the two groups (90 HC and 45 OS).

Supplementary Data Sheet S7 The different degrees at the class level (p-value) between the two groups (90 HC and 45 OS).

Supplementary Data Sheet S8 The different degrees at the order level (p-value) between the two groups (90 HC and 45 OS).

Supplementary Data Sheet S9 The different degrees at the family level (p-value) between the two groups (90 HC and 45 OS).

Supplementary Data Sheet S10 The specific bacterial taxa and predominant bacteria associated with OS (n = 45) and HC (n = 90) by LEfSe method.

Supplementary Data Sheet S11 The corresponding LDA value and p-value of the significantly different genera in the two groups (90 HC and 45 OS).

Supplementary Data Sheet S12 The corresponding LDA value and p-value of microbial community gene function for samples in the two groups (90 HC and 45 OS).

Supplementary Data Sheet S13 The p-value of Spearman’s correlation analysis between oral microbiome and clinical data of OS.

Supplementary Data Sheet S14 The corresponding output value of each optimal microbial marker in the train set by random forest classifier model (60 HC and 30 OS).

Supplementary Data Sheet S15 The corresponding POD value of each sample in the train set (60 HC and 30 OS).

Supplementary Data Sheet S16 The corresponding output value of each optimal microbial marker in the test set by random forest classifier model (30 HC and 15 OS).

Supplementary Data Sheet S17 The corresponding POD value of each sample in the test set (30 HC and 15 OS).
