# Supplementary material for: Oral microbiota distinguishes patients with osteosarcoma from healthy controls
Source: Front Cell Infect Microbiol. 2024 Jul 11;14:1383878. doi: 10.3389/fcimb.2024.1383878 (PMC11269967; doi:10.3389/fcimb.2024.1383878)
Supplement: Supplementary file 2 [file DataSheet_2.docx]

**Additional file 1 Figure S1-6 and legends**


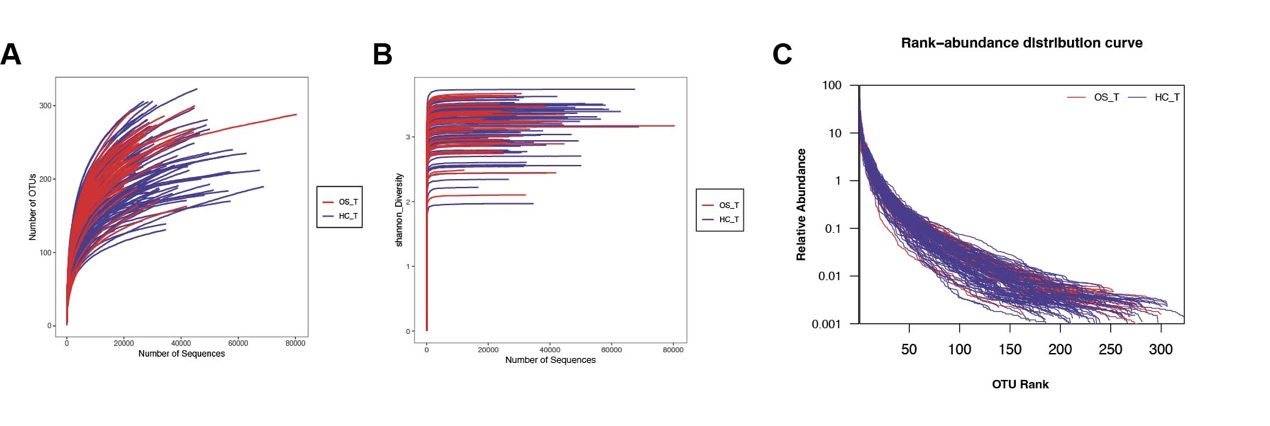


**Figure S1. Results of rarefaction analysis.**

A. A rarefaction curve between the number of OTUs and the number of sequences in OS (n = 45) and HC (n = 90).

B. A Shannon-wiener curve between the number of sequences and the Shannon diversity in OS (n = 45) and HC (n = 90).

C. A rank-abundance distribution curve for the OTUs of OS (n = 45) and HC (n = 90).

OS, osteosarcoma; HC, healthy control; OTU, operational taxonomic unit.


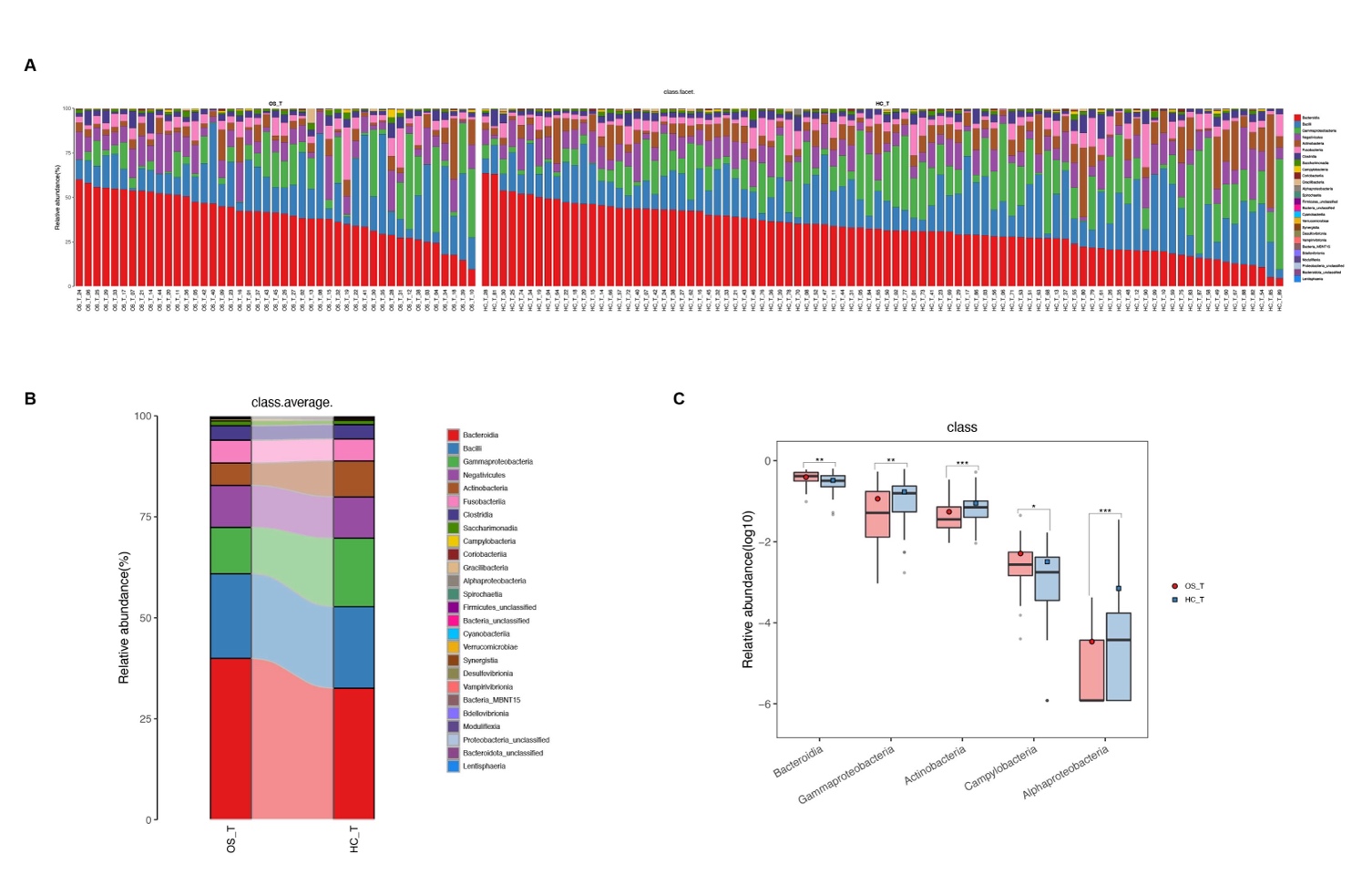


**Figure S2**

A. The composition and abundance of bacterial community in each sample at the class level (45 OS and 90 HC).

B. The average composition and relative abundance of the bacterial community at the class level in both groups (45 OS and 90 HC).

C. At the class level, two bacterial populations were significantly enriched, and three bacterial populations were significantly reduced in OS (n = 45) versus HC (n = 90).

*, p<0.05, **, p<0.01, ***, p<0.001; OS, osteosarcoma; HC, healthy controls


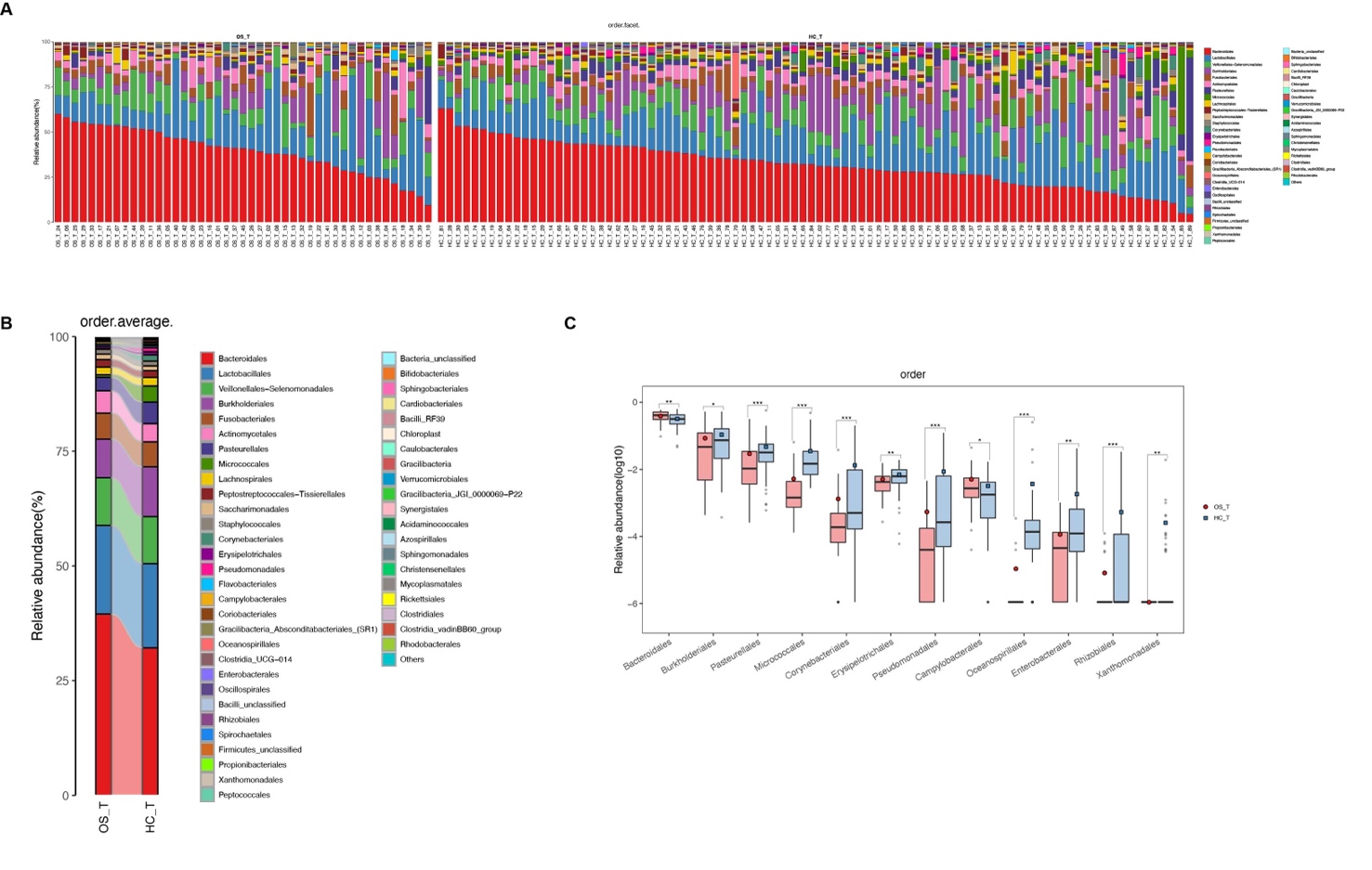


**Figure S3**

A. The composition and abundance of bacterial community in each sample at the order level (45 OS and 90 HC).

B. The average composition and relative abundance of the bacterial community at the order level in both groups (45 OS and 90 HC).

C. At the order level, two bacterial populations were significantly enriched, and ten bacterial populations were significantly reduced in OS (n = 45) versus HC (n = 90).

*, p<0.05, **, p<0.01, ***, p<0.001; OS, osteosarcoma; HC, healthy controls

**
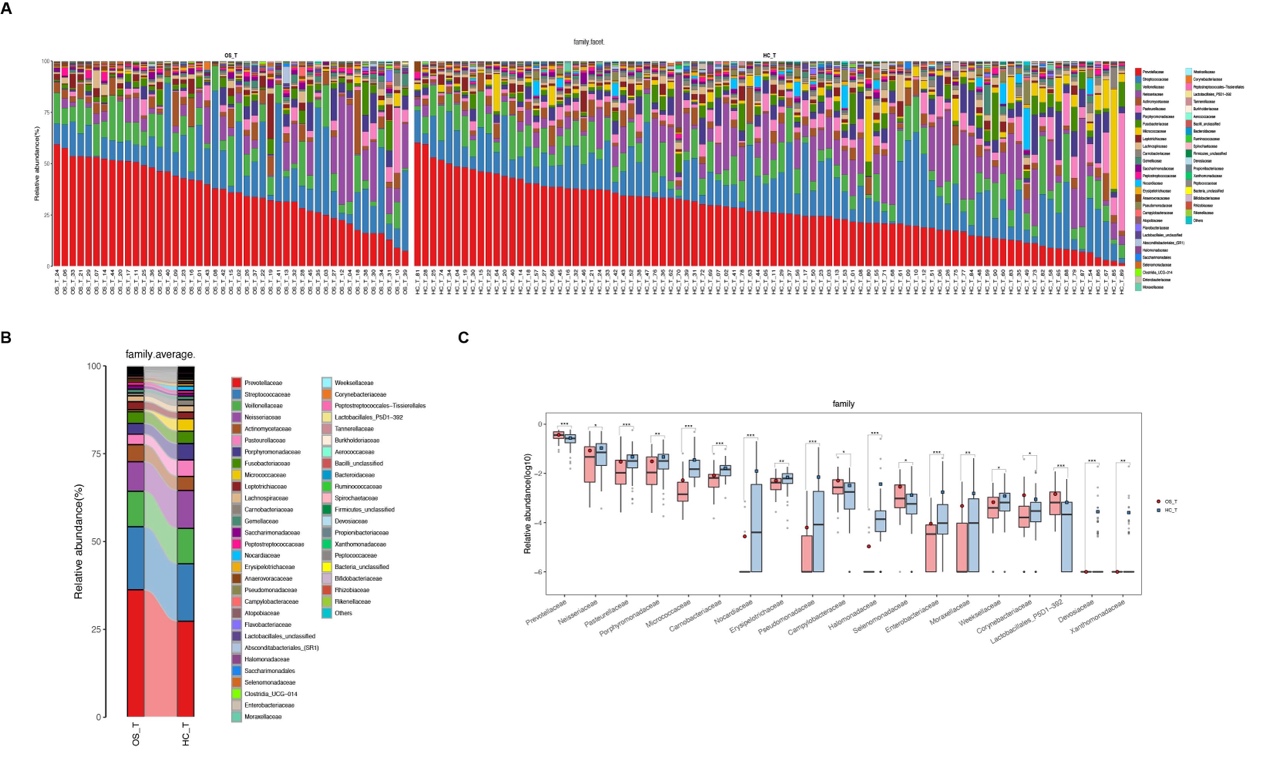
**

**Figure S4**

A. The composition and abundance of bacterial community in each sample at the family level (45 OS and 90 HC).

B. The average composition and relative abundance of the bacterial community at the family level in both groups (45 OS and 90 HC).

C. At the family level, two bacterial populations were significantly enriched, and ten bacterial populations were significantly reduced in OS (n = 45) versus HC (n = 90).

*, p<0.05, **, p<0.01, ***, p<0.001; OS, osteosarcoma; HC, healthy controls


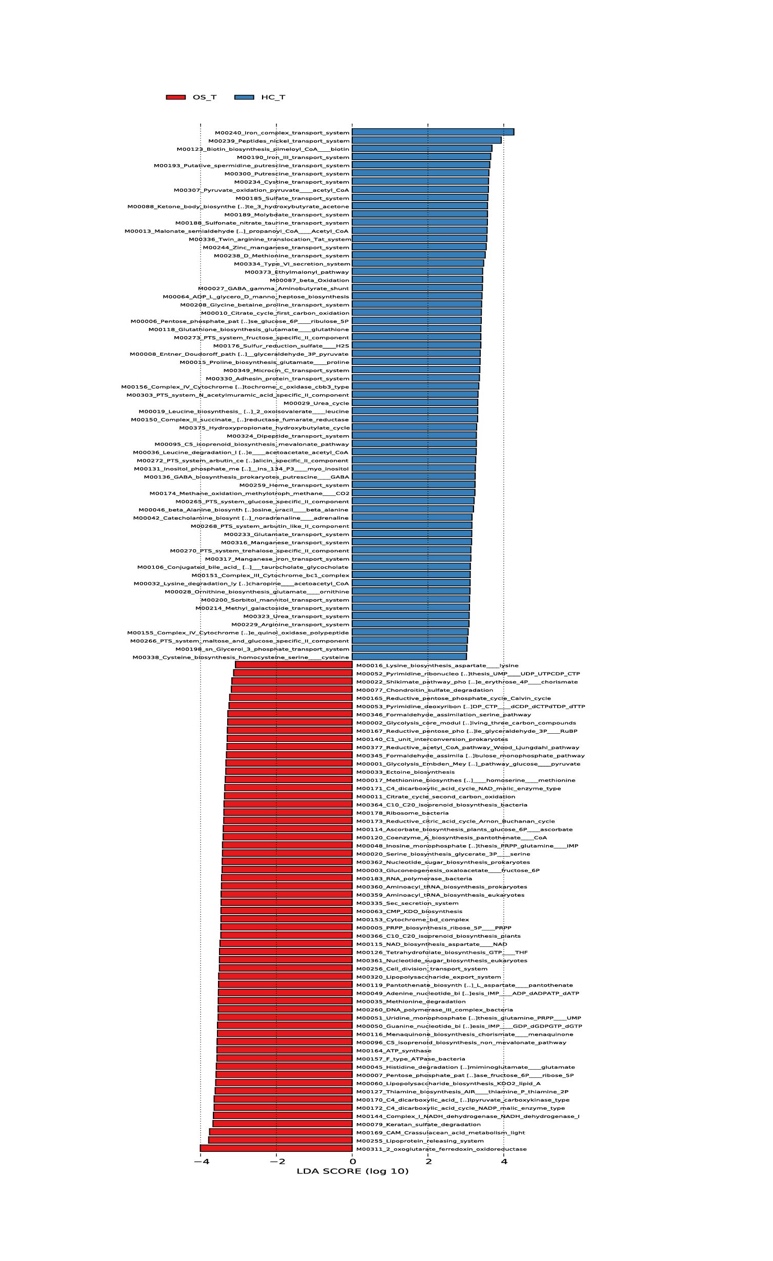


**Figure S5**

LDA score showed the reletive alterations of key functional and metabolic modules between OS (n = 45) and HC (n = 90).

OS, osteosarcoma; HC, healthy controls


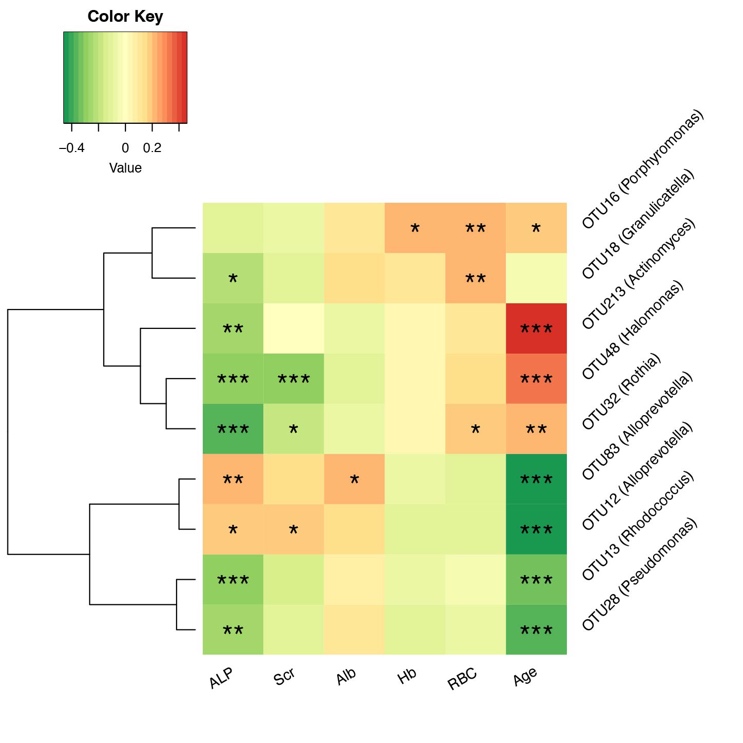


**Figure S6**

Heatmap showed the partial Spearman’s correlation coefficients between 9 OTUs and 6 clinical indicators in OS patients (n=45).

OTU, operational taxonomic unit; RBC, red blood cell count; Hb, hemoglobulin; ALB,

albumin; Scr, serum creatinine; ALP, alkaline phosphatase.
